# Peer Review of “COVID-19 Pneumonia Diagnosis Using Medical Images: Deep Learning-Based Transfer Learning Approach”

**DOI:** 10.2196/83236

**Published:** 2025-09-26

**Authors:** Emmanuel Ndezure

**Affiliations:** 1 Kwame Nkrumah University of Science and Technology, Kumasi, Ghana

**Keywords:** computer vision, COVID-19 pneumonia diagnosis, deep learning, transfer learning, medical imaging analysis

*This is the peer-review report for “COVID-19 Pneumonia Diagnosis Using Medical Images: Deep Learning-Based Transfer Learning Approach.”*

## Round 1 Review

### General Comments

This manuscript [1] investigates the application of deep learning, particularly transfer learning using VGG16, VGG19, and ResNet-50, for diagnosing COVID-19 through computed tomography and and X-ray images. The topic is important and timely, especially considering the enduring threat of COVID-19 variants and the burden on global health care systems. The author demonstrates technical familiarity with deep learning techniques, model tuning, and performance evaluation. However, there are areas where the study could be improved to enhance its rigor, clarity, and impact.

### Specific Comments

#### Major Comments

1. Dataset description and bias: the paper mentions using a dataset of 6259 images (4651 COVID-19 cases and 1608 normal cases). However, there is no discussion on potential biases in the dataset, such as the source of the images, demographic diversity (age, gender, and geographic location), or the balance between COVID-19 and normal cases. Addressing these aspects would strengthen the validity of the results. I suggest that the author include a detailed description of the dataset, including sources, demographic information, and steps taken to mitigate bias, and consider discussing the imbalance in the dataset and how it might affect model performance.

2. Comparative analysis with existing methods: while the paper reports high accuracy (97.73%) for the proposed models, it lacks a comprehensive comparison with other state-of-the-art methods or baseline models. This makes it difficult to assess the novelty and superiority of the proposed approach. I suggest that the author add a comparative table or section that contrasts the performance of VGG16, VGG19, and ResNet-50 with other recent studies or baseline models and highlight the unique contributions of this work.

3. Clinical relevance and practical deployment: the study focuses on technical performance metrics but does not discuss the clinical applicability of the models. For instance, how would these models integrate into real-world health care settings? What are the potential challenges (eg, computational resources, interpretability for clinicians)? I suggest that the author expand the discussion on clinical relevance, including limitations and practical considerations for deployment in health care systems.

4. Language and grammar: the manuscript needs extensive language editing. There are frequent grammatical issues, awkward phrasing (eg, “the 1608 belong to healthy people”), and repetition. A professional edit is highly recommended to improve readability and flow.

5. Figures and tables: several figures (eg, confusion matrices, loss/accuracy curves) are referenced but lack sufficient clarity, labeling, or captions. Figures 4 to 8 must be embedded clearly within the results discussion and interpreted to guide the reader. Ensure figures are high resolution and correctly formatted.

6. Overstatement of results: the paper claims high performance (97.73% accuracy), yet offers little discussion on external validity or overfitting risks. Since cross-validation was performed on a relatively small dataset, these results may not generalize well. The author should tone down claims and discuss limitations.

7. Dataset description and ethics: while the dataset is described as publicly available, the manuscript lacks ethical approval or justification. Clarify whether ethical clearance was required. Also, organize the dataset description into a single, detailed section including data sources, balance between classes, preprocessing applied, and augmentation steps.

8. Evaluation metrics and statistical rigor: the paper heavily relies on accuracy, sensitivity, specificity, and *F*_1_-score, but fails to report CIs or conduct statistical tests to validate performance differences between models. Including receiver operating characteristic area under the curve values and visualizations would also strengthen the evaluation.

9. Novelty and contribution not clearly established: while the paper uses popular convolutional neural network architectures, there is no clear indication of what is novel in this study compared to the extensive body of existing work using these same models on similar datasets. What distinguishes this work? Is it the dataset size, preprocessing technique, tuning strategy, or model ensemble?

#### Minor Comments

10. Hyperparameter tuning details: the paper describes hyperparameter tuning but does not explain the rationale behind the selected ranges (eg, learning rate and batch size). A brief justification for these choices would improve reproducibility. I suggest adding a sentence or two explaining why the specified ranges for hyperparameters were chosen.

11. Use consistent terminology throughout (eg, “deep learning model” versus “CNN-based model”).

12. Data augmentation techniques: these are described generically. Specify which augmentations were applied and how frequently. Were augmentation parameters validated?

13. Please structure the abstract under clear headings, Background, Objective, Methods, Results, and Conclusion, to aid clear reading and comprehension.

## Round 2 Review

### Specific Comments

#### Major Comments

I commend the author for the comprehensive revisions made in response to the initial review. The manuscript now demonstrates significant improvements in clarity, organization, and scientific rigor. Key concerns, including dataset bias, comparative evaluation with existing methods, clinical applicability, language quality, and statistical robustness, have been adequately addressed. Figures and tables, which were initially submitted as separate files, have now been properly embedded and contextualized within the main manuscript, greatly improving readability and interpretation of results.

All my previous comments have been satisfactorily responded to, and I have no further critical concerns. I find the revised manuscript suitable for publication.
